# Exogenous sodium diethyldithiocarbamate, a Jasmonic acid biosynthesis inhibitor, induced resistance to powdery mildew in wheat

**DOI:** 10.1002/pld3.212

**Published:** 2020-04-09

**Authors:** Yinghui Li, Lina Qiu, Qiang Zhang, Xiangxi Zhuansun, Huifang Li, Xin Chen, Tamar Krugman, Qixin Sun, Chaojie Xie

**Affiliations:** ^1^ Key Laboratory of Crop Heterosis and Utilization (MOE) and State Key Laboratory for Agrobiotechnology Beijing Key Laboratory of Crop Genetic Improvement China Agricultural University Beijing China; ^2^ Institute of Evolution University of Haifa, Mt. Carmel Haifa Israel

**Keywords:** auxin, brassinosteroid, jasmonic acid, powdery mildew, resistance, sodium diethyldithiocarbamate, wheat

## Abstract

Jasmonic acid (JA) is an important plant hormone associated with plant–pathogen defense. To study the role of JA in plant–fungal interactions, we applied a JA biosynthesis inhibitor, sodium diethyldithiocarbamate (DIECA), on wheat leaves. Our results showed that application of 10 mM DIECA 0–2 days before inoculation effectively induced resistance to powdery mildew (*Bgt*) in wheat. Transcriptome analysis identified 364 up‐regulated and 68 down‐regulated differentially expressed genes (DEGs) in DIECA‐treated leaves compared with water‐treated leaves. Gene ontology (GO) enrichment analysis of the DEGs revealed important GO terms and pathways, in particular, response to growth hormones, activity of glutathione metabolism (e.g., glutathione transferase activity), oxalate oxidase, and chitinase activity. Gene annotaion revealed that some *pathogenesis‐related* (*PR*) genes, such as *PR1.1*, *PR1*, *PR10*, *PR4a*, *Chitinase 8*, *beta‐1,3‐glucanase*, *RPM1*, *RGA2*, and *HSP70,* were induced by DIECA treatment. DIECA reduced JA and auxin (IAA) levels, while increased brassinosteroid, glutathione, and ROS lesions in wheat leaves, which corroborated with the transcriptional changes. Our results suggest that DIECA can be applied to increase plant immunity and reduce the severity of *Bgt* disease in wheat fields.

## 1 INTRODUCTION

Bread wheat (*Triticum aestivum* L.) is one of the most important food crops worldwide. Powdery mildew, caused by *Blumeria graminis f*. sp. *tritici* (*Bgt*), is an obligate biotrophic ascomycete fungus that invades the aerial parts of wheat and can cause large yield losses ranging from 30% to 40% under heavy epidemics (Mehta, 2014; Singh et al., 2016). Deployment of resistance (*R*) genes is currently one of the most economical and sustainable methods for disease control. However, the rapidly evolving wheat powdery mildew fungus can escape the recognition by some *R* genes (Dangl, Horvath, & Staskawicz, 2013; Jones & Dangl, 2006). Understanding the molecular basis of plant innate or induced defense responses will enable to find new methods for disease control.

During the long history of co‐evolution with pathogens, plants have developed a multifaceted innate immunity system. After the recognition of pathogen invasion, several downstream signaling events are elicited in the plant cell, including influx of Ca^2+^ into the cytosol, reactive oxygen species (ROS) accumulation, and transient activation of mitogen‐activated protein kinases (MAPK) signaling cascades (Boller & Felix, 2009; Choudhury, Rivero, Blumwald, & Mittler, 2017; Tsuda & Katagiri, 2010). Plant hormones act as immune signals, triggering extensive transcriptional reprogramming, and resulting in an efficient defense response (Bari & Jones, 2009). These plant hormones include salicylic acid (SA) and its methylated derivative MeSA (Park, Kaimoyo, Kumar, Mosher, & Klessig, 2007), and jasmonic acid (JA) and its methylated derivative MeJA (Browse, 2009; Truman, Bennett, Kubigsteltig, Turnbull, & Grant, 2007; Wu, Wang, & Baldwin, 2008), auxin (Truman, Bennett, Turnbull, & Grant, 2010), and brassinosteroids (BRs) (Yu, Zhao, & He, 2018).

JA is involved in the defense against necrotrophic pathogens, preventing plant cell death and inducing defense responses to restrict further pathogen infection (Singh, Singh, Gautam, & Nandi, 2019). Treatment with JA is shown to protect plants against herbivore attack and reduce the severity of infection by necrotrophic fungi (Baldwin, 1998; Thomma, Eggermont, Broekaert, & Cammue, 2000; Zalewski et al., 2019). JA signaling also plays an important role in mediating plant defense against some biotrophic or hemibiotrophic pathogens (De Vleesschauwer, Gheysen, & Höfte, 2013; Yan & Xie, 2015; Zalewski et al., 2019). Exogenous application of MeJA up‐regulates some defense genes and results in efficient reduction of disease development (Desmond et al., 2005; Thomma et al., 2000; Wasternack, 2007; Xu et al., 1994). However, contradictory evidences have been published regarding the role of JA in *Bgt* resistance in wheat. Duan et al., (2014), show that exogenous MeJA significantly enhance *Bgt* resistance in susceptible wheat varieties, while Xiang et al., (2011), show that MeJA application does not induce resistance to *Bgt* in wheat. Therefore, the role of JA in plant–fungal interactions is still not clear.

Crosstalk of plant hormones is important for disease resistance, for example, previous reports show an antagonistic relationship between the JA and SA signaling pathways in plant–fungal interactions. SA can mediate programmed cell death response in plant cells and restrict (hemi) biotrophic pathogens to the infection site, preventing pathogen proliferation (An & Mou, 2011; Nishimura & Dangl, 2010). Exogenous treatments with SA (or SA analogs) have been shown to induce resistance against (hemi) biotrophic pathogens in several plant species (Görlach et al., 1996; Van Wees, De Swart, Van Pelt, Van Loon, & Pieterse, 2000). However, (hemi) biotrophic pathogenic bacteria can activate plant JA signaling to dampen SA signaling and facilitate host colonization (Patkar et al., 2015). Necrotrophic pathogens manipulate SA‐JA antagonism to suppress JA‐mediated defense (El Oirdi et al., 2011; Rahman, Oirdi, Gonzalez‐Lamothe, & Bouarab, 2012). In addition, it has been shown that auxin (indole‐3‐acetic acid/IAA) is a signaling molecule that can promote pathogens infection (Bielach, Hrtyan, & Tognetti, 2017; Chen et al., 2007; McClerklin et al., 2018; Navarro et al., 2006), and brassinolide (BL) can induce resistance to several pathogens in plants (Deng et al., 2016; Nakashita et al., 2003).

Sodium diethyldithiocarbamate (DIECA) has been used as a JA biosynthesis inhibitor in plants and likely inhibits the JA pathway by shunting 13(S)‐hydroperoxylinolenic acid to 13‐hydroxylinolenic, thereby sharply reducing the precursor pool leading to cyclization and eventual synthesis of JA (Farmer, Caldelari, Pearce, Walker‐Simmons, & Ryan, 1994). Application of DIECA has been shown to significantly reduce JA levels in multiple plant species and reduce the expression of some resistance gene, such as *TaJRLL1* and *PR3* (Hu & Zhong, 2008; Hu, Neill, Cai, & Tang, 2003; Xiang et al., 2011). However, there is no clear and solid report for fungal resistance imposed by different regulated JA levels in wheat. Our results showed that application of DIECA, the inhibitor of JA biosynthesis, could induce resistance to *Bgt* in wheat, while exogenous MeJA did not. In addition to inhibition of JA after DIECA application, the level of IAA was decreased and brassinosteroid (BR) was increased, and accumulation of glutathione and ROS was observed. These findings corroborated with wide transcriptional regulation induced by DIECA, for example, the up‐expression of *PR* genes and enriched GO terms such as response to growth hormones, activity of glutathione metabolism, oxalate oxidase, and chitinase activity. Moreover, our results suggested that DIECA application can be used to control *Bgt* in the field.

## 2 MATERIALS AND METHODS

### 2.1 Plant materials

The following powdery mildew (*Bgt* isolate E09) susceptible cultivars were used: Xuezao, Chinese Spring, Liaochun18, Liaochun10, and Fielder; and barley cultivar Golden promise (susceptible to barley powdery mildew isolate A6).

*Arabidopsis* lines (Col wild‐type and the *eds1* and *pad4* mutants) were provided by Dr. Zhaorong Hu, China Agriculture University.

### 2.2 Pathogen maintenance and inoculation

*Bgt* isolate E09 was provided by Prof. Xiayu Duan, Institute of Plant Protection, Chinese Academy of Agricultural Sciences. Barley powdery mildew (*Bgh*) isolate A6 and *Arabidopsis* powdery mildew *G. cichoracearum* strain UCSC1 were provided by Prof. Qianhua Shen. Isolate E09 was maintained on the susceptible wheat line Xuezao through weekly transfer to new plants. One‐week‐old wheat plants were used for both spray and spot inoculations. Wheat plants were inoculated at a density of 100–150 conidia/mm^2^ using a blowing machine in a vaccination tower. Infection types (IT) were classified into six classes in accordance with a previous study with IT 0–4 representing no visible symptoms (0), necrotic flecks (0;), highly resistant (1), resistant (2), susceptible (3), and highly susceptible (4) reactions, respectively (Liu, Sun, Ni, Yang, & McIntosh, 1999). *G. cichoracearum* strain UCSC1 was maintained by growing it on *pad4* mutants. Four‐week‐old *Arabidopsis* plants were inoculated using the same methods as those used for inoculation of wheat with strain E09. About 16 plants in each treatment were used for phenotyping, and the representative leaves or plants were used for photograph.

### 2.3 Coomassie blue staining

For microscopic observations of fungal development, at 24–120 hpi, the *Bgt*‐infected leaves’ segments were collected for coomassie blue staining as described by Li et al., (2016). For microscopic observations, leaf segments (5 cm in length) were stored in 50% glycerol and examined under an Olympus BX‐43 microscope (Olympus Corporation).

The germination and penetration rates of conidiophores (number of germinated spores and penetrated spores relative to the total number of spores, respectively) were visualized after staining with Coomassie blue. Germinated spores show germinated germ tubes; penetrated spores show developed hyphae and have initiated the formation of young colonies. In each independent experiment, 15–20 leaf segments were observed at 24, 48, 96, and 120 hpi. Microscopic measurements were used for calculating the mean of germination and penetration rates, using three independent replicated experiments. Statistical significance was determined by paired Student's *t* test.

### 2.4 DIECA treatments

*DIECA Treatment at seedling stage (1)*—DIECA in sterile water containing 0.02% Silwett‐L77 (Fisher Scientific, Cat. NC0138454) was used as treatments, while water (0.02% Silwett‐L77) was used as control treatment. To select the optimal concentration to be used in the spraying experiment, we applied a preliminary test of DIECA spraying of 0, 0.1, 1, 5, 10, 20, and 30 mM, at seedling stage (when the first leaf was fully expanded). Subsequently, the 5 mM and 10 mM DIECA treatments were selected. DIECA was sprayed onto wheat leaves at seedling stage until the liquid was dripping off the leaves.

*Frequency and duration of DIECA treatments*—To evaluate the optimal timing of DIECA treatment for effective enhancement of disease resistance, 10 mM DIECA was applied at different time durations, prior to or after powdery mildew inoculation. With continuous powdery mildew inoculation in the greenhouse, plants were sprayed (10 mM DIECA) every 2–6 days.

*DIECA treatment on Bgt*‐*infected detached leaf segments (2)*—Pretreatment included 5 mM and 10 mM DIECA and water, smeared on detached wheat leaf segments placed on agar plates (1% agar, containing 0.05% benzimidazole, SIGMA). One day after pretreatment, the segments were inoculated using the pathogen inoculation method described above.

*DIECA treatment in the field (3)—*Plants at the adult stage (flowering stage) were sprayed with water, 1 mM DIECA, 5 mM DIECA, or 10 mM DIECA, or were untreated. DIECA or water was sprayed twice (every five days) prior to the outbreak of powdery mildew disease. Two wheat field treatments were used: (a) Artificial field inoculation‐in which Xuezao seedlings with *Bgt* sporulation were transplanted as spreader, in a field containing uninfected Xuezao seedlings. (b) Naturally infected field‐in a field located 30–50 m away from the artificial inoculation field.

### 2.5 MeJA treatments

The water and 0.2, 0.5, and 1 mM MeJA solutions contained 0.02% Silwett‐L77 were prepared. MeJA was dissolved in the Silwett‐L77 and mixed with water. In the first two days, Xuezao plants were sprayed once a day; at the third day, plants were infected with *Bgt E*09.

### 2.6 RNA extraction, CDNA library construction, RNA‐SEQ, and data analysis

Leaves for RNA extraction were samples at seedling stage (*DIECA* treatment 1). The treatments of water or DIECA were applied once a day for 2 days, and on the third day, leaf samples were collected. Ten leaves of each pot were pooled together, as one biological repetition for RNA extraction. Three biological repetitions were employed for each treatment. Total RNA was extracted using RNA pure Plant Kit (TIANGEN). cDNA Libraries were generated using the NEB Next UltraTM RNA Library Prep Kit for Illumina (NEB) following the manufacturer's recommendations. Paired‐end reads were generated on IlluminaHiseq 2500 platform. Sequencing data were analyzed by using BMKCloud (http://en.biocloud.net/). Adaptor sequences and low‐quality sequence reads were removed from the data sets. Tophat2 tools were used to map the reads to the wheat reference genome (IWGSC RefSeq Annotation v1.0). Only reads with perfect match or one mismatch were further analyzed and annotated based on the reference genome. Gene function was annotated based on the following databases: Nr (NCBI non‐redundant protein sequences); Nt (NCBI non‐redundant nucleotide sequences); Pfam (Protein family); KOG/COG (Clusters of Orthologous Groups of proteins); Swiss‐Prot (A manually annotated and reviewed protein sequence database); KO (KEGG Ortholog database); and GO. Differential expression analysis of the two groups was performed using the DESeq R package (1.10.1). Transcripts with an adjusted *p*‐value ≤ .05 and with |log2 fold change| ≥ 2 found by DESeq were assigned as differentially expressed. GO enrichment analysis of the DEGs was implemented using the GOseq R package based on the Wallenius non‐central hyper‐geometric distribution (Young, Wakefield, Smyth, & Oshlack, 2010), which can adjust for gene length bias in DEGs. We used KOBAS (Mao, Cai, Olyarchuk, & Wei, 2005) software to test for statistically significant enrichment of DEGs in KEGG pathways.

### 2.7 Measurements of endogenous fatty acids, glutathione, plant hormones, and DAB staining for ROS

Application of 10 mM DIECA was used once a day for two days (DIECA treatment 1). On the third day, leaf samples were collected for the measurements of endogenous fatty acids, glutathione, and plant hormones. Fatty acid analysis was measured as described by Li et al., (2016), using HP6890 gas chromatograph (Agilent Technologies). Leaf tissues were dried in an oven at 45℃ for 60 hr and then ground into a powder; 200 mg of powder for each sample was placed in a screw capped glass vial. Glutathione and plant hormones, including JA, IAA, SA, BR, gibberellins (GA3 and GA4), dihydrozeatin riboside (DHZR), zeatin riboside (ZR), indolepropionic acid (IPA), and abscisic and acid (ABA), were measured from leaf tissues as described by Cao, Li, Chen, Liu, and Li (2016), and Zhao et al., (2006), with slightly modification  by using different internal reference and antibodies. Leaf cell death response was observed at 7 dpi by trypan blue staining as described previously (Koch & Slusarenko, 1990). ROS was estimated using DAB staining solution (0.1 g DAB, 100 ml distilled water, KOH adjusted to pH = 5.8) to stain infected leaves for 8 hr at 28℃ and then 100% ethanol was used to depigment infected leaves for 1 day.

## 3 RESULTS

### 3.1 DIECA application induced powdery mildew resistance in wheat

**FIGURE 1:**
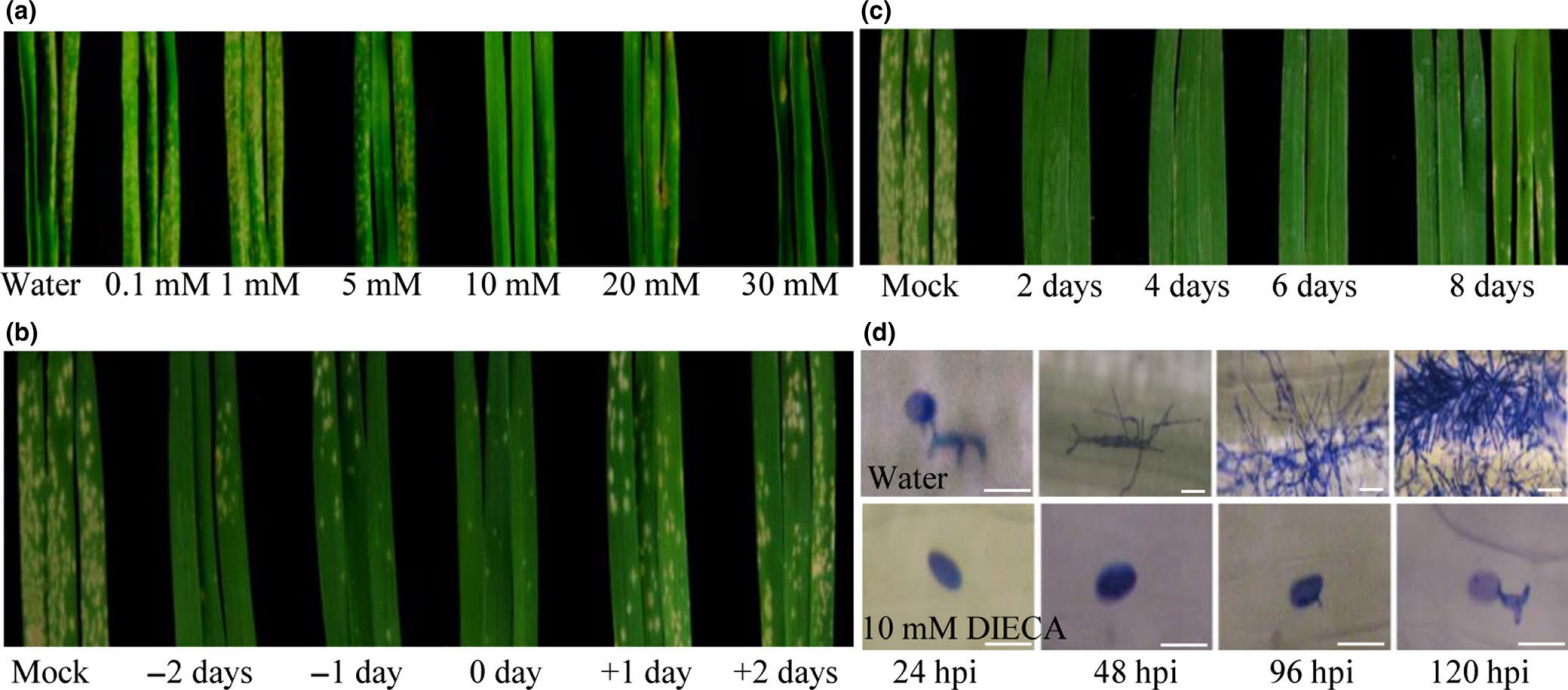
DIECA induced resistance to *Bgt* in wheat. (a) Resistance phenotypes of Xuezao leaves treated with water and different concentrations of DIECA (0.1, 1, 5, 10, 20, and 30 mM) at 7 days post‐*Bgt* infection (dpi). The infection type (IT) of water, 0.1 and 1mM DIECA‐treated plants is 4; 5 mM DIECA (IT = 2); 10–30 mM DIECA (IT = 0 or 0;). (b) Resistance phenotypes of Xuezao leaves with 10 mM DIECA applied at different times before and after *Bgt* inoculation. (−2 to −2) DIECA treatments were performed at 0, 1, and 2 days before (−) and after (+) *Bgt* inoculation. Mock (IT = 4); −2 and −1 days (IT = 1); 0 day (IT = 0); and +1 and +2 days (IT = 3) (c) Resistance phenotypes (7 dpi) of plants treated with 10 mM DIECA at differing frequencies. Plants were treated with 10 mM DIECA once every 2, 4, 6, or 8 days; three leaves are shown for the treatments performed every 2, 4, and 6 days treatment, where all leaves were higly resistant to *Bgt* (IT = 0). Six leaves are shown for the DIECA treatment performed every 8 days because although the old leaves were resistant, some of the new leaves started be infected with *Bgt*. (d) Morphology of conidiophores in water‐treated and DIECA‐treated Xuezao leaves at 24, 48, 96, and 120 hpi. Bars = 50 µm

Preliminary test of different DIECA concentrations indicated that plants were more resistant to *Bgt* with increased concentrations (Figure 1a). On the contrary, application of 0.2–1 mM MeJA could not induce *Bgt* resistance in wheat (Figure S1). Consequently, 10 mM of DIECA treatment that induced effective resistance to *Bgt*, including small necrosis response without any visible disease symptoms, was selected for further analysis. Application of DIECA 0–2 days prior to *Bgt* inoculation effectively induced disease resistance, while application of DIECA 1–2 days after inoculation was not effective (Figure 1b). With continuous *Bgt* inoculation in the greenhouse, spraying once every 2–6 days was required for inducing durable resistance. Lower and new leaves started to be infected with *Bgt* when DIECA was applied once every 8 days (Figure 1c). The best treatments were obtained when 10 mM DIECA was sprayed one day prior to *Bgt* infection, and was further treated every 4 days in order to induce durable and effective resistance.

Microscopic observation showed that germination and penetration rates of *Bgt* on DIECA‐treated leaves were remarkably lower than in untreated or water‐treated leaves at 24, 48, 96, and 120 hr post‐infection (hpi) (Table S1; Figure 1d). Even after removal of DIECA from the leaves using sterile water before *Bgt* infection, plants were highly resistant to *Bgt* (Figure S2). The specificity of the treatment in four different susceptible wheat cultivars (Chinese Spring, Liaochun 18, Liaochun 10, and Fielder) showed that *Bgt* resistance was enhanced in all the four wheat cultivars (Figure S3). Moreover, 5 mM and 10 mM DIECA application induced barley resistance to *B. graminis* f. sp. *hordei* (*Bgh*) (Figure S4) and also enhanced powdery mildew *Golovinomyces cichoracearum* UCSC1 resistance in *Arabidopsis* (Figure S5).

### 3.2 Transcriptome analysis of wheat response to DIECA

Resistance to *Bgt* was observed only when plants were treated by DIECA prior to inoculation. Therefore, we used uninoculated DIECA‐treated and water‐treated plants for transcriptome analysis by RNA‐seq. A total of 170,850,209 clean reads were obtained, with ≥25,749,518 clean reads in each pool (i.e., one cDNA library prepared from six samples). For each sample, ≥89.47% of the reads had a quality score of Q30 (Table S2). Following assembly, 82.63%–84.26% of the sequences in each library could be mapped to the Chinese Spring wheat genome reference sequence (Tables S3). In total, 118,189 distinct assembled unigenes were annotated after blast searches against several databases (Table S4). Differentially expressed genes (DEGs) between the DIECA and the water‐treated libraries were identified using the following criteria: |log_2_ fold change| ≥ 2 (*p* ≤ .05). A total of 432 DEGs were identified, of which 364 were up‐regulated in treated plants while 68 were down‐regulated (Tables S5 and S6). DEGs identified in different biological replicates were clustered together in a heat map of expression levels, indicating good reproducibility between replicates (Figure S6).

**FIGURE 2:**
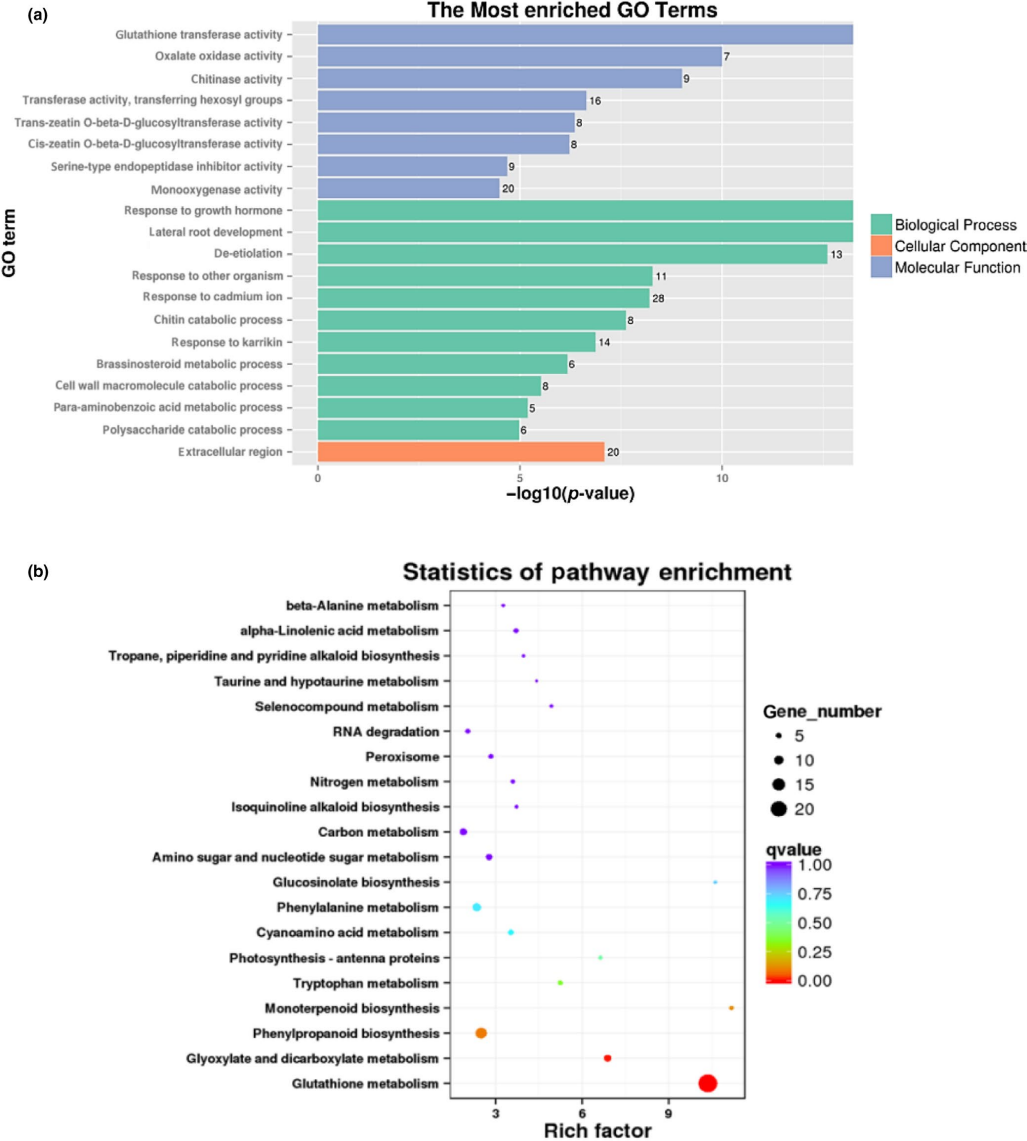
The top 20 enriched GO terms and KEGG pathways. (a) GO enrichment analysis of genes differentially expressed in response to DIECA. Data are presented according to the *p*‐value. The names of the 20 most highly enriched GO terms are arranged on the vertical axis according to the ‐log 10 (*p*‐value); the horizontal axis represents the ‐log 10 (*p*‐value). (b) Statistical scatter plot showing the pathways enriched in genes differentially regulated in response to DIECA. The color represents the Q value as shown in the legend. Q values are the *p* values corrected for multiple hypothesis testing and range from 0 to 1. The closer the Q value is to zero, the more significant the enrichment. The horizontal axis indicates the rich factor, where higher a rich factor indicates a greater degree of enrichment. The size of each circle indicates the number of DEGs in that pathway

The highly enriched GO terms of molecular function included glutathione transferase activity, oxalate oxidase activity, and chitinase activity. The highly enriched GO term of biological process included response to growth hormone, lateral root development, and de‐etiolation (Figure 2a). KEGG enrichment analysis showed that the highly enriched pathways were glutathione metabolism, glyoxylate and dicarboxylate metabolism, phenylpropanoid biosynthesis, monoterpenoid biosynthesis, tryptophan metabolism, photosynthesis‐antenna proteins, and α‐linolenic acid metabolism (Figure 2b). GO and KEGG enrichment analyses showed that pathways most highly enriched in the DEGs were associated with glutathione metabolism and growth hormones (Figure 2).

Among the up‐regulated DEGs in DIECA‐treated leaves we identified pathogenesis‐related genes, including genes encoding PR1.1, PR1, PR10, PR4a, disease resistance protein RGA3, chitinase 8, beta‐1,3‐glucanase, beta‐glucosidase 31, peroxidase 1, peroxidase 2, peroxidase 3, heat shock factor A4e, disease resistance protein RPM1, disease resistance protein RGA2, and HSP70 (Table S5). In addition, genes encoding glutathione transferase and glutathione S‐transferase (2.5.1.18), which function in the glutathione metabolism pathway (Ko00480), were induced (Figure S7). In the α‐linolenic acid metabolism pathway (Ko00592), the 1.3.1.42 (encoding 12‐oxophytodienoic acid reductase) was also induced by DIECA (Figure S8). Obviously, many of the up‐regulated DEGs have been previously reported to be involved in the plant immune response to pathogens. The down‐regulated DEGs in DIECA‐treated leaves were mainly photosynthesis‐related genes, such as chlorophyll a/b‐binding protein, chlorophyll a/b‐binding protein WCAB precursor, and NADPH‐dependent diflavin oxidoreductase 1 (Table S6).

### 3.3 Glutathione, fatty acids, and plant hormones changed with the DIECA application

#### 3.3.1 Glutathione and fatty acids

**FIGURE 3:**
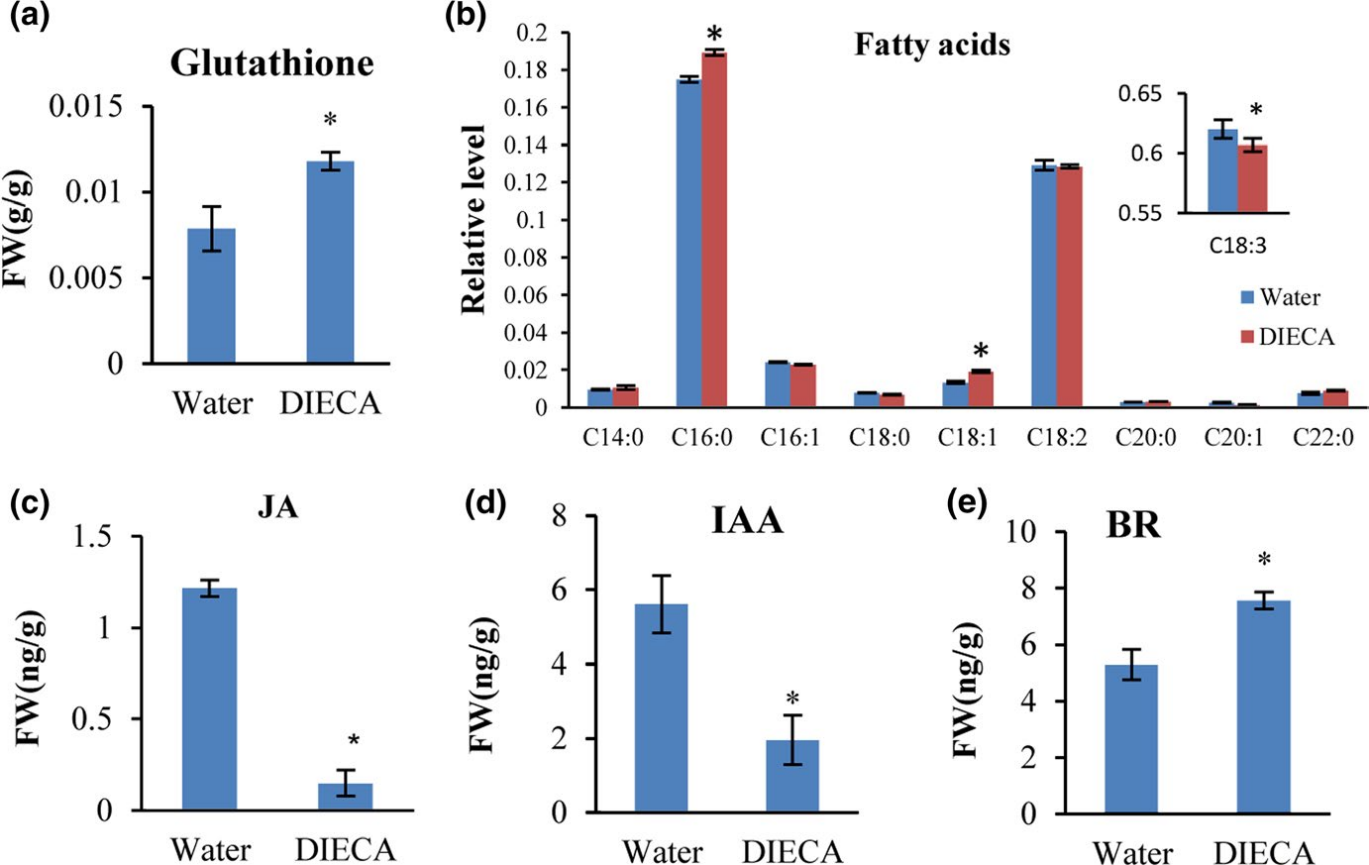
Content of glutathione (a), fatty acids (b), JA (c), IAA (d), and BR (e) levels in water‐treated and DIECA‐treated Xuezao leaves. Each value is the mean ± *SE* of three independent biological repetitions. Asterisks indicate a significant difference from the water‐treated mock control at *p* ≤ .05 determined by Student's *t* test

The analysis indicated that glutathione level was significantly increased following DIECA application (Figure 3a). JA and its derivatives are lipid‐derived hormones synthesized from linolenic acid (Bari & Jones, 2009; Wasternack & Hause, 2013). We found that DIECA application resulted in slightly higher levels of C16:0 and C18:1, and slightly lower level of C18:3, while the levels of other fatty acids (C14:0, C16:1, C18:0, C18:2, C20:0, C20:1, and C22:0) did not changed by DIECA application (Figure 3b). These results were supported by KEGG enrichment analysis which showed that glutathione and α‐linolenic acid metabolism pathways were enriched in DEGs.

#### 3.3.2 Plant hormones

We analyzed the levels of JA and other nine additional plant hormones in the water or DIECA‐treated wheat leaves. Our results showed that JA level was decreased by ~88%, in addition, IAA level was decreased by ~65%, and BR level was increased by ~42.8% in the DIECA‐treated leaves as compared with the water‐treated leaves (Figure 3c, 3d and 3e). No change was observed in other analyzed plant hormones, including SA, GA4, GA3, DHZR, ZR, IPA, and ABA (Figure S9).

Previous studies showed that SA pathways might synergistically interact with JA or IAA pathways (Patkar et al., 2015; Robert‐Seilaniantz, Navarro, Bari, & Jones, 2007; Yuan, Liu, & Lu, 2017); therefore, DIECA was applied on two *pad4* and *eds1* mutants, which have loss of function of SA‐mediated resistance pathways. The results indicated that powdery mildew resistance to UCSC1 was still induced (Figure S10). This suggests that the induced resistance by DIECA might be independent of the SA pathway.

### 3.4 DIECA induced cell death and ROS accumulation

**FIGURE 4:**
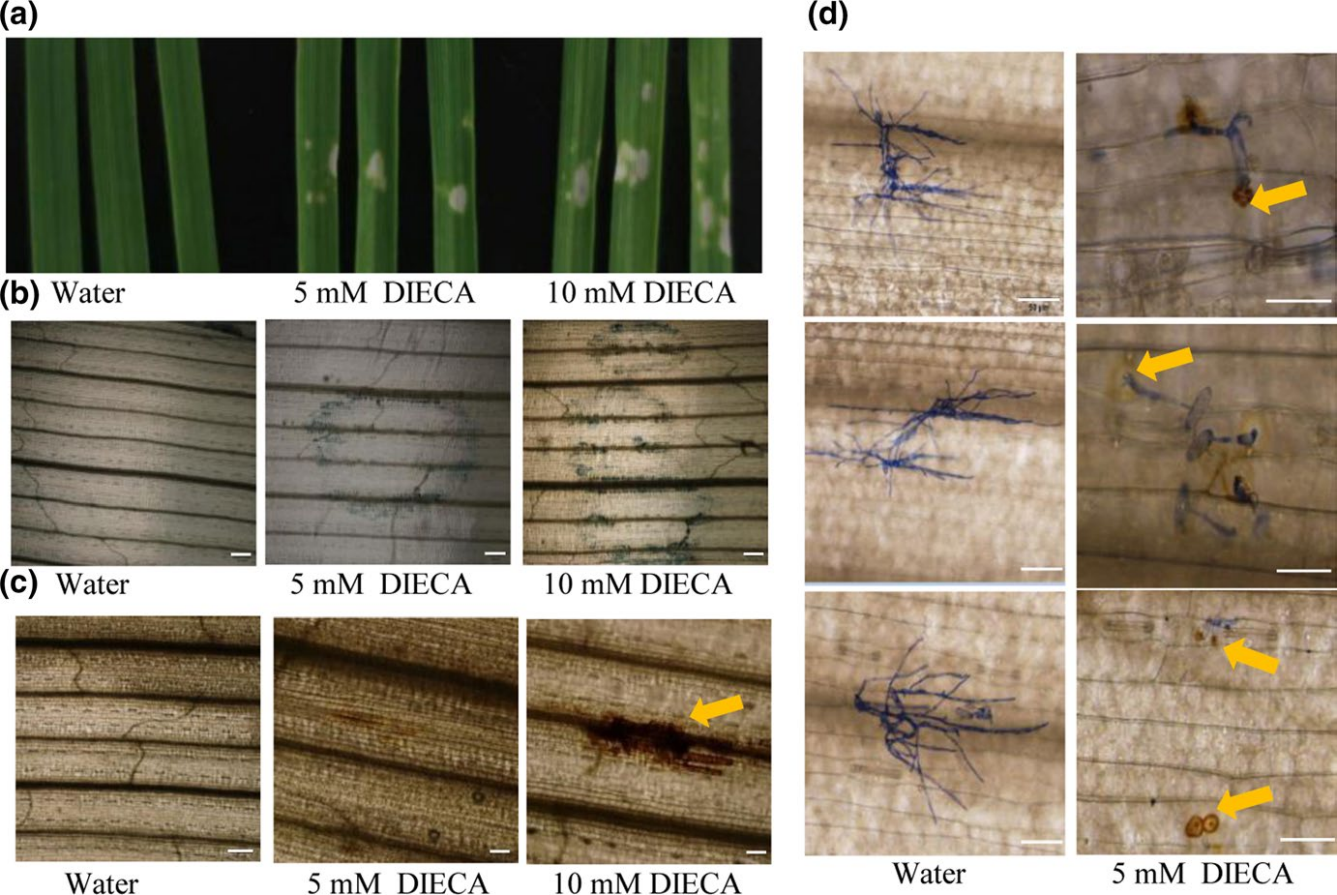
DIECA induced cell death and ROS accumulation. (a) Morphology of Xuezao leaves at 10 days post‐application of water, 5 mM DIECA, or 10 mM DIECA without *Bgt* infection. Trypan blue (b) and DAB (c) staining of Xuezao leaves at 10 days post‐application of 5 mM DIECA or 10 mM DIECA without *Bgt* infection. (d) DAB and Coomassie blue staining of Golden promise leaves pretreated with water or 10 mM DIECA, at 3 dpi. ROS accumulation is indicated by yellow arrows. Bars = 50 µm

We could find visible spontaneous lesions on central parts of some wheat leaves 1 week after 5 mM or 10 mM DIECA application in the absence of *Bgt* infection (Figure 4a). This damage might be caused by concentrated remains of DIECA on the leaves associated with cell death and ROS accumulation (Figure 4b and c). Nevertheless, in infected plants, ROS accumulation also could be found around the site of infection, with hyphae growth arrest, while no ROS accumulation was observed in the water‐treated leaves, with visible hyphae growth (Figure 4d). The ROS accumulation and cell death observed on DIECA‐treated plants might also contribute resistance to powdery mildew.

### 3.5 Wheat powdery mildew resistance under field conditions

**FIGURE 5:**
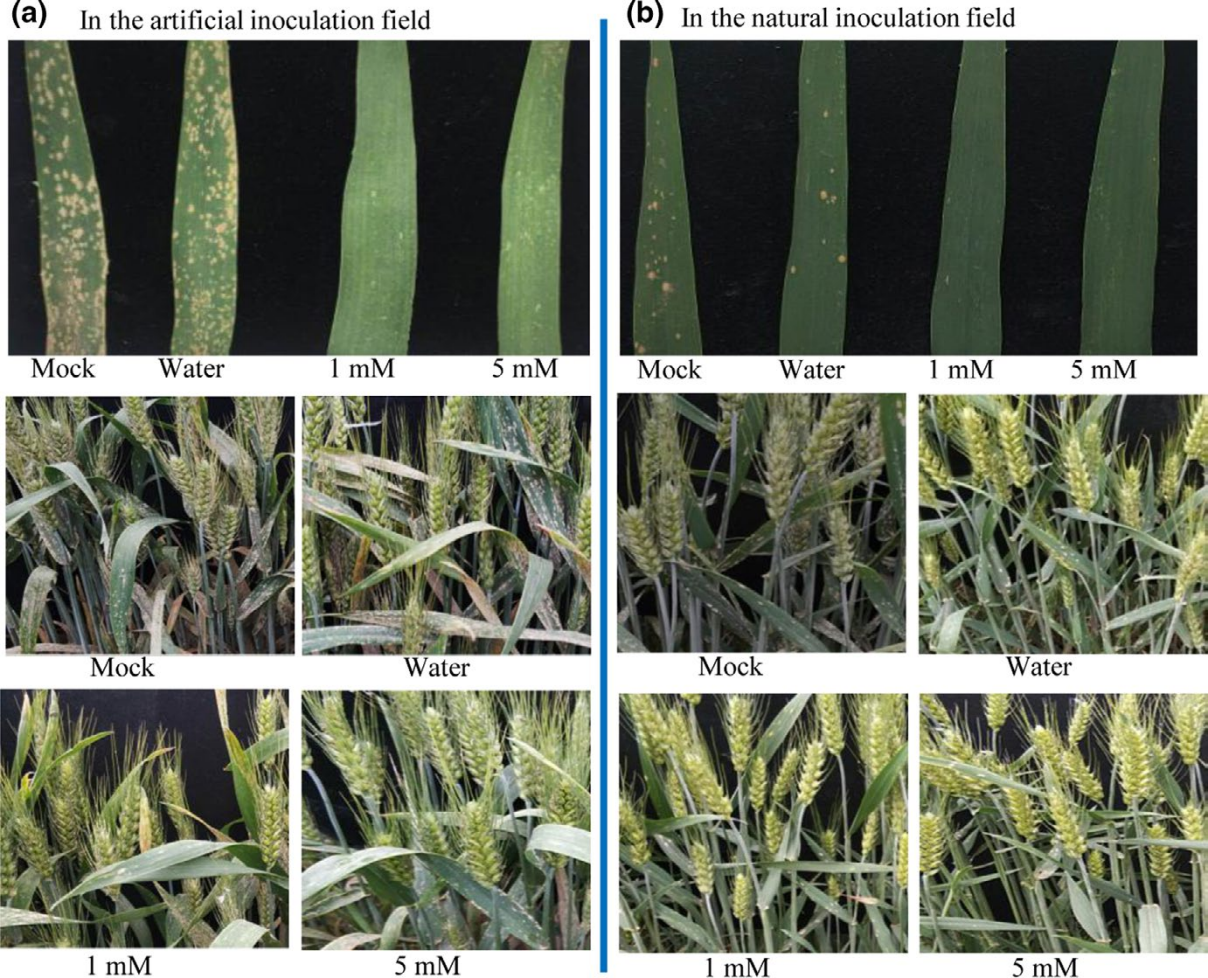
DIECA application induced wheat powdery mildew resistance under field conditions. Images of untreated, water‐treated, and 1 mM and 5 mM DIECA‐treated Xuezao plants in the artificial inoculation field (a) and natural infection field (b) at 5 days after the second application of water or DIECA. 2018, in Beijing

DIECA application experiments were performed under field conditions in two years, 2017 and 2018. The results of 2017 showed that 5 mM and 10 mM DIECA induced high level of resistance to *Bgt* in the artificial inoculation field. However, since 10 mM DIECA caused necrotic spots on wheat leaves (Figure S11), the 1 mM and 5 mM DIECA treatments were selected for experiments at artificial inoculation and a natural infection field in 2018. Both 1 mM and 5 mM DIECA application induced effective resistance in these two locations (Figure 5a and b).

## 4 DISCUSSION

Plant hormones crosstalk mediate complex signal transduction networks, involve in different defense strategies to pathogens (Li, Han, Feng, Yuan, & Huang, 2019). In the current study, we showed that the inhibition of JA biosynthesis by DIECA triggered hormonal alteration and transcriptional reprogramming involved in plant defense. Furthermore, we demonstrated that the application of DIECA prior to inoculation with the pathogen induced powdery mildew resistance in wheat, barley, and *Arabidopsis*. The inhibition of JA induced a remarkable reduction of IAA and increase of BR levels, while no change was detected in level of SA, which was shown to have a role in plant resistance and decrease auxin and JA levels in previous studies (Yuan et al., 2017). Changes were not observed in the levels of other tested plant hormones, including GA, ZR, DHZR, IPA, and ABA. Many reports indicate that JA is an important plant hormone associated with plant resistance. In some cases, down‐regulation in JA pathway increases disease resistance, as is previously shown in mutants deficient in JA signaling, which are more resistant to *Pst* DC3000 (Kloek et al., 2001; Thines et al., 2007). Other indications show that exogenous application of MeJA up‐regulates some defense genes, accompanied by efficient reduction of disease development. For example, MeJA induces the *PR‐1b* and *osmotin* (*PR‐5*) mRNA accumulation in tobacco, also cause efficient reduction of disease development by *Alternaria brassicicola*, *Botrytis cinerea,* and *Plectosphaerella cucumerina* in *Arabidopsis*, and delays symptom development by the crown rot pathogen *Fusarium pseudograminearum* in wheat (Desmond et al., 2005; Thomma et al., 2000; Wasternack, 2007; Xu et al., 1994). Our results showed that pretreatment with MeJA did not induce effective resistance to *Bgt* in wheat, while application of JA biosynthesis inhibitor DIECA could induce highly resistance to *Bgt* in wheat (Figure 1 and Figure S1).

We observed a reduction of IAA level following DIECA treatment (Figure 3d). It was shown that IAA can promote some biotrophic pathogen diseases (Wang & Fu, 2011). For example, exogenous application of IAA aggravates *Xanthomonas oryzae* pv. *oryzae* and *X. oryzae* pv. *oryzicola* disease progression (Ding et al., 2008; Fu et al., 2011). Exogenous application of naphthaleneacetic acid (NAA) increases maize vulnerability to *F. graminearum* infection and indirectly suppresses plant immunity by reducing the levels of SA and JA (Ye et al., 2019). *Arabidopsis* mutant *axr2‐1,* which is insensitive to auxin, displays a 10‐fold reduction in *P. syringae* pv. *Maculicola* growth compared with wild‐type plants (Nagpal et al., 2000; Timpte, Wilson, & Estelle, 1994; Wang, Pajerowska‐Mukhtar, Culler, & Dong, 2007). Thus, blocking the auxin pathway improves plant resistance. Several studies report that JA and auxin share some signaling pathway components, JA‐mediated processes might be upstream of the auxin biosynthetic pathways and auxin might have a role in regulating JA level (Cai et al., 2014; Hentrich et al., 2013; Tiryaki & Staswick, 2002; Williams, Fernández‐Calvo, Colinas, Pauwels, & Goossens, 2019).

The observed induction of BR in our study corroborates with earlier studies showing that BR plays critical roles in the regulation of plant growth and development as well as responses to biotic and abiotic stress factors (Figure 3e; Peres et al., 2019). BR applications seem to exert an effect on immunity to a wide array of pathogens in different plant species, such as enhancing resistance to *Fusarium* infection in barely (Ali, Kumar, Khan, & Doohan, 2013) and inducing resistance to powdery mildew in tobacco (Nakashita et al., 2003). Other evidence shows that BR has negative roles in plant resistance in rice, and JA‐mediated defense can suppress the BR‐mediated susceptibility to infection rice black‐streaked dwarf virus, suggesting an antagonistic relationship between BR and JA effects in viral defense (He et al., 2017).

The transcriptome analysis results indicated that the most highly enriched GO terms among the DEGs included response to growth hormone (Figure 2a). Indeed, the observed increase of BR level together with reduction of JA and IAA, after DIECA treatment, might contribute to wheat resistance to *Bgt* (Figure 3). However, the crosstalk of IAA and JA and BR have been reported to regulate plant resistance and growth (He et al., 2017; Peres et al., 2019; Zhou, Song, & Xue, 2013). Among these three hormones, JA plays an important role in plant growth inhibition (Huang, Liu, Liu, & Song, 2017), and BR is growth‐promoting hormone (Lozano‐Durán & Zipfel, 2015). Our result showed that DIECA treatment reduced the growth of Arabidopsis (Figure S5), and slightly reduction was also observed in wheat (Figure S2). Since JA was reduced and BR was increased (i.e., both promoting growth), we suggest that the cause of growth reduction might be via the reduction of IAA after DIECA application (Figure 3). Further studies are needed to determine which of the three hormones (JA, BR, or IAA) is the key regulator, or a synergistic effect of the three hormones is the important factor for inducing *Bgt* resistance in wheat.

The results of transcriptome analysis indicated that “glutathione transferase activity” was the most highly enriched GO term (e.g., KEGG enrichment analysis; Figure 2b) among the up‐regulated DEGs after DIECA application. These genes encoding glutathione transferase and glutathione S‐transferase (GST, 2.5.1.18), which function in the glutathione metabolism pathway (Ko00480), were among the up‐regulated DEGs after DIECA application (Figure S7). In some cases, *GSTs* have been shown to contribute to resistance against powdery mildew (Gullner, Komives, Király, & Schröder, 2018). The *GstA1* gene is specifically induced by fungal infection (Mauch & Dudler, 1993). *GST* is also required for resistance against *O. neolycopersici* in tomato (Pei et al., 2011). Glutathione has also been reported to be an important molecule for plant–pathogen resistance. The γ‐glutamylcysteine synthetase mutant *pad2‐1* contains only about 22% of the wild‐type amount of glutathione and is highly susceptible to oomycete pathogen *Phytophthora brassicae*; feeding mutant plants glutathione can restore the glutathione level and resistance to the pathogen (Parisy et al., 2007). In addition to glutathione generation, we have identified ROS accumulation in wheat leaves in DIECA‐treated leaves (Figure 4c and d). ROS have been postulated to be an integral part of the plant defense response, acting as local and systemic signal molecules that are involved in the activation of antimicrobial defenses (Waszczak, Carmody, & Kangasjärvi, 2018). Furthermore, transcriptome analysis indicates that DIECA induced genes encoding peroxidase 1, peroxidase 2, and peroxidase 3, which have been reported to play a significant role in generating H_2_O_2_ during the plant defense response and in conferring resistance to a wide range of pathogens (Bindschedler et al., 2006). In plant–pathogen interactions, ROS participate in a coordinated way in regulating the hypersensitive response (Bellin, Asai, Delledonne, & Yoshioka, 2013). Thus, ROS and glutathione accumulation, identified in the current study, may be an important part of DIECA‐induced resistance mechanism to *Bgt* in wheat.

The downstream genes of pathways in plant disease resistance may be involved in these biological processes, especially inducing the expression of some *PR* genes, including *PR1*, *PR2*, *chitinase* (*PR3*, *PR8*, and *PR11*), *peroxidase* (*PR9*), and *oxalate oxidase* (*PR15* and *PR 16*). Most of those *PR* genes can be used as potential candidate genes for improvement of the pathogen resistance of wheat and barley (Wang et al., 2018). For example, *PR* genes encoding hydrolytic enzymes chitinases and β‐1,3‐glucanases are very important in plants for invading pathogen, and overexpression of some *PR* genes improve the resistance to pathogens in plants (Ali et al., 2018; Ebrahim, Usha, & Singh, 2011). It suggested that those DIECA‐induced *PR* genes, such as encoding PR1.1, PR1, PR10, PR4a, chitinase 8, beta‐1,3‐glucanase, and beta‐glucosidase 31, might contribute to resistance to powdery mildew in wheat, and those *PR* genes could be used as candidate genes for improving wheat resistance (Table S5).

From agricultural point of view, our results clearly showed that spraying with DIECA 0–2 days prior to *Bgt* inoculation induced effective resistance in wheat. Our results also show that DIECA did not have a direct toxic effect on the growth of *Bgt* hyphae (Figure 1b). Furthermore, when DIECA‐pretreated wheat leaves were washed before *Bgt* infection, leaves were still highly resistant (Figure S2). The results of our two‐year field experiments showed that DIECA application in wheat fields could significantly reduce the severity of powdery mildew disease (Figure 5). Unlike in the greenhouse experiments where 10 mM DIECA was the appropriate concentration for inducing resistance, 1 or 5 mM DIECA could effectively be used to control *Bgt* in field during heading date of wheat.

## 5 CONCLUDING REMARKS

By implementing integral metabolic, transcriptomic and plant pathology methods, we show here that inhibition of JA biosynthesis led to alteration in IAA and BR which triggered the accumulation of ROS and glutathione and up‐regulation of *pathogenesis‐related* genes. Eventually inducing defense responses to *Bgt* in Wheat. Our results indicate that spraying with DIECA can be used to control *Bgt* in the field.

## CONFLICT OF INTEREST

The authors declare that the research was conducted in the absence of any commercial or financial relationships that could be construed as a potential conflict of interest.

## AUTHOR CONTRIBUTIONS

CX, QS, YL, and LQ conceived the project. YL and LQ performed most of the experiments. QZ, XZ, HL, and XC provided help for experiments. YL wrote the manuscript. LQ, TK, and CX improved and revised this manuscript.

## Supporting information

Figure S1‐S11

Table S1

Table S2

Table S3

Table S4

Table S5

Table S6

Reviewers202028
