# Supplementary material for: Exogenous sodium diethyldithiocarbamate, a Jasmonic acid biosynthesis inhibitor, induced resistance to powdery mildew in wheat
Source: Plant Direct. 2020 Apr 9;4(4):e00212. doi: 10.1002/pld3.212 (PMC7146025; doi:10.1002/pld3.212)
Supplement: Supplementary file 1 — Figure S1‐S11 [file PLD3-4-e00212-s001.pdf]

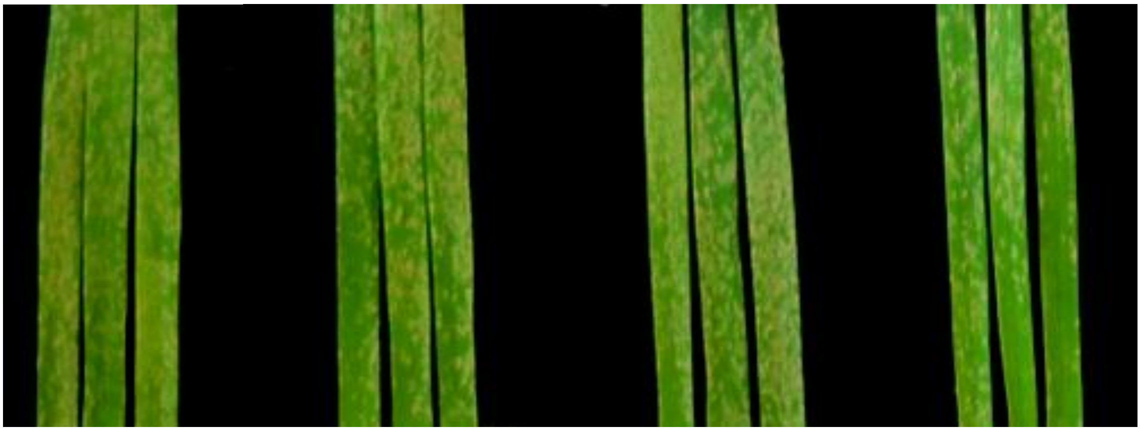

Water 0.2 mM MeJA 0.5 mM MeJA 1 mM MeJA

Figure S1 Morphology and resistance phenotypes of Xuezao leaves, pretreated with water, 0.2 mM MeJA, 0.5 mM MeJA, or 1 mM MeJA, at 7 dpi with *Bgt*.

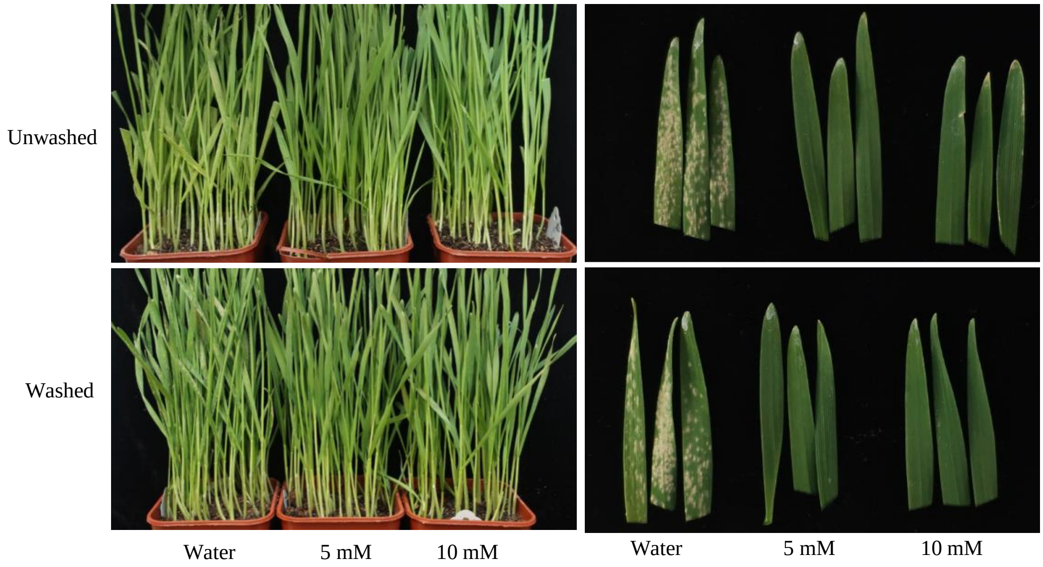

Figure S2 Resistance phenotypes of unwashed (above panels) or washed (below panels) wheat leaves pretreated with water, 5 mM DIECA, or 10 mM DIECA, at 7 dpi. The washed leaves were gently washed with sterile water before *Bgt*-infection.

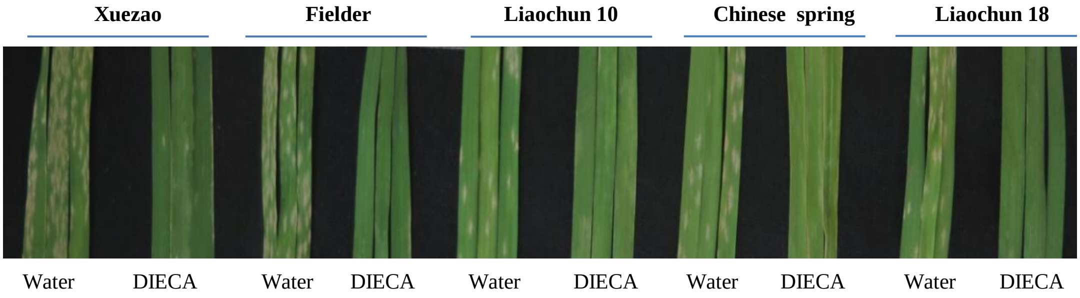

Figure S3 Resistance phenotypes of several wheat varieties, pretreated with 10 mM DIECA, at 7 dpi with *Bgt*.

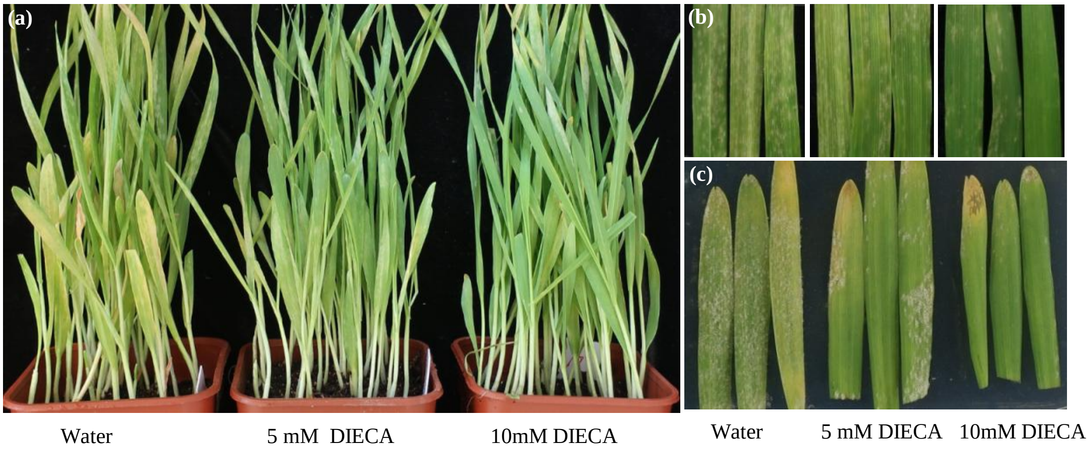

Figure S4 Resistance phenotypes of barley (Golden promise), pretreated with water, 5 mM DIECA, or 10 mM DIECA, at 7 dpi with *Bgh*. **(a)** The resistance phenotypes of whole plants, sprayed with water, 5 mM DIECA, or 10 mM DIECA, at 7 dpi. **(b)** The resistance phenotypes of Golden promise leaves, sprayed with water, 5 mM DIECA, or 10 mM DIECA, at 7 dpi. **(c)** The resistance phenotypes of detached leaves, smeared with water, 5 mM DIECA, or 10 mM DIECA and incubated on 1% agar, at 7 dpi.

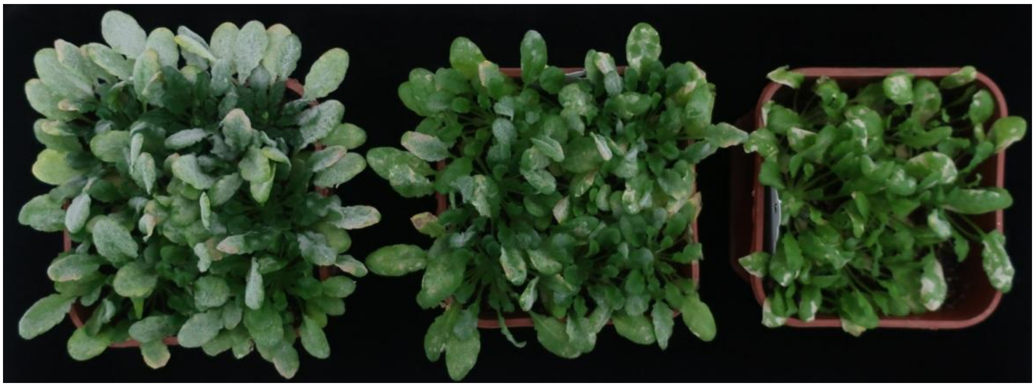

Water 5 mM DIECA 10 mM DIECA

Figure S5 Resistance phenotypes of *Arabidopsis*, pretreated with water, 5 mM DIECA, or 10 mM DIECA, at 10 dpi with *Golovinomyces cichoracearum* UCSC1.

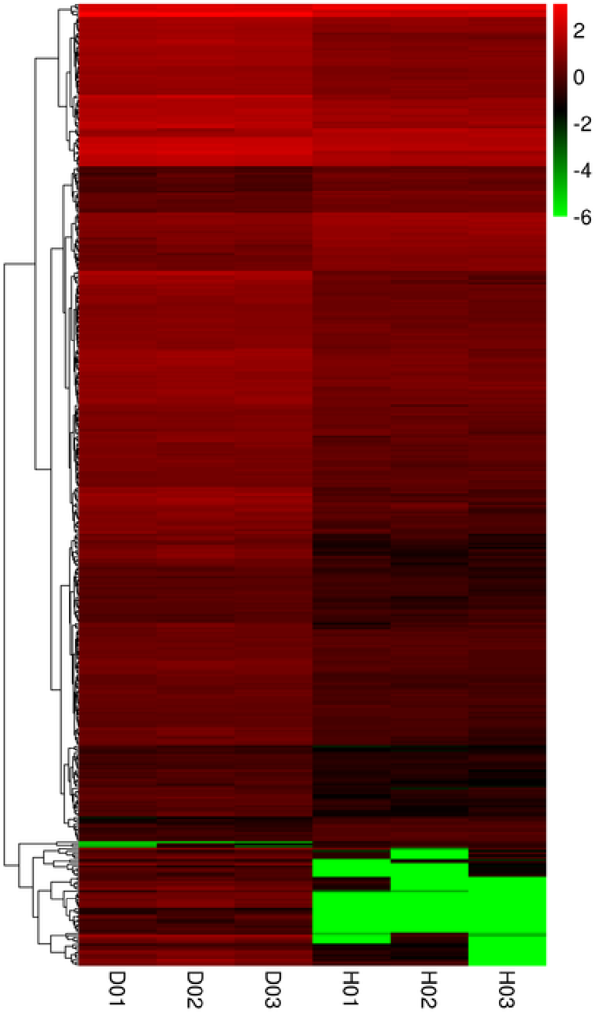

Figure S6 Cluster analysis of DEG expression levels. D01, D02, D03: three biological repeats of the 10 mM DIECA treatment; H01, H02, H03: three biological repeats of the water treatment. The color represents the expression level (absolute intensity), where red indicates a higher expression level and green indicates a lower expression level.

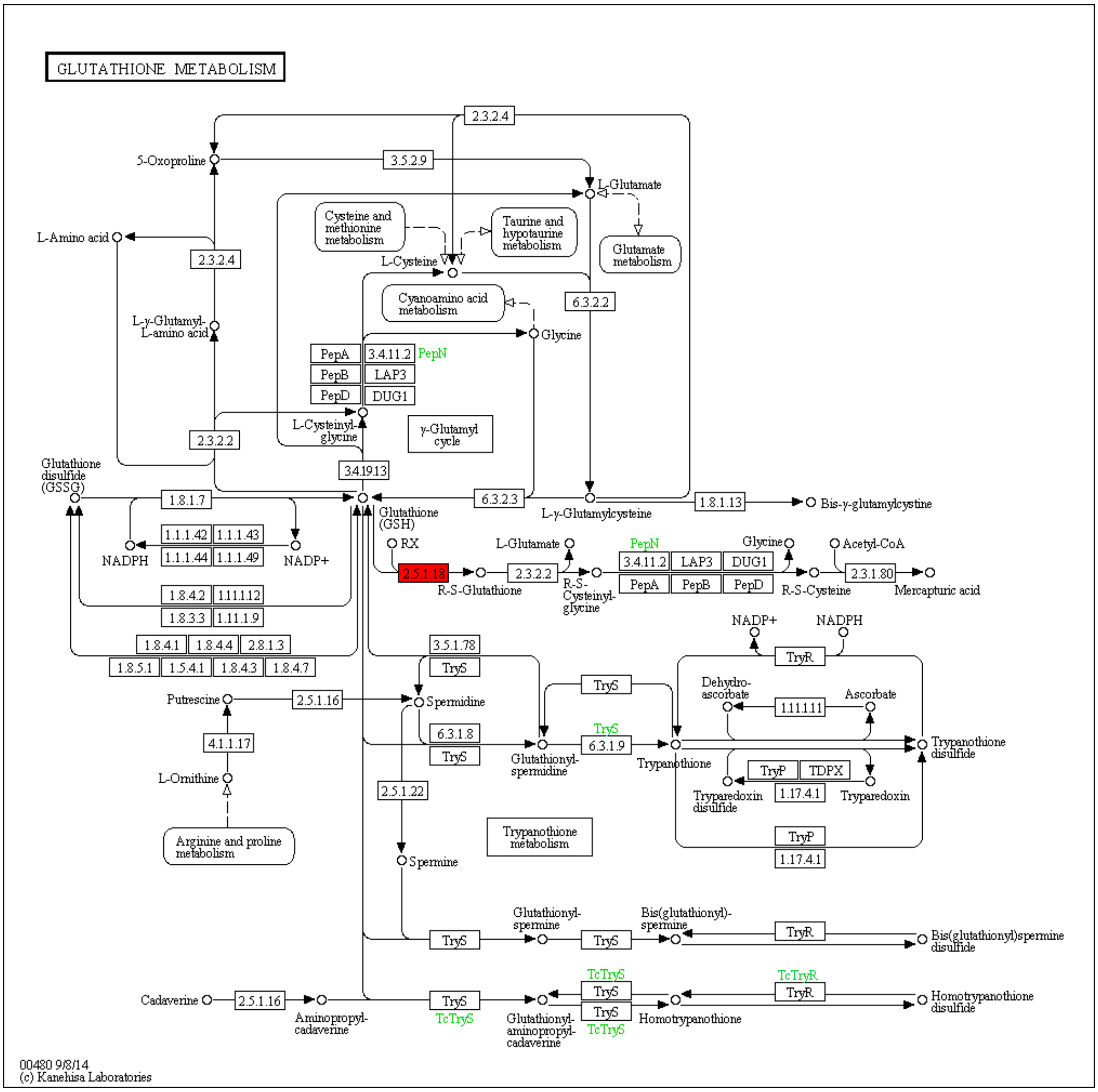

Figure S7 The expression of genes in the glutathione metabolism pathway (Ko00480) changed in response to DIECA application. The red color of 2.5.1.18 indicates that the expression of these genes was induced by DIECA application.

(2.5.1.18):TraesCS1A01G186100;TraesCS1A01G186300;TraesCS1A01G186500;TraesCS1A01G186600;TraesCS1A01G186700;TraesCS1A01G187000;TraesCS1B01G113700;TraesCS1B01G194100;TraesCS1B01G194300;TraesCS1B01G194700;TraesCS1B01G194900;TraesCS1D01G095400;TraesCS1D01G190100;TraesCS1D01G191100;TraesCS2A01G218700;TraesCS4A01G104300;TraesCS4B01G044500;TraesCS4B01G199700;TraesCS4B01G330900;TraesCS4D01G200700;TraesCS5D01G030000;TraesCS6D01G127700;TraesCS6D01G127800;TraesCS7A01G477900
(2.36086885216201;5.92182674638854;3.55942647311078;6.12629157034251;2.48392618701104;4.34248422083662;1.08917560925506;2.11541136412485;1.78321884582547;1.76688197715042;4.20292338565029;1.97949365805918;3.49896321499985;3.41801938419329;1.49608329538136;5.52769571737121;1.57485734407127;7.67902182341009;2.44454694019847;4.97144595544271;1.69102703150392;4.10007796651527;2.80305495418261;1.23252080985183)

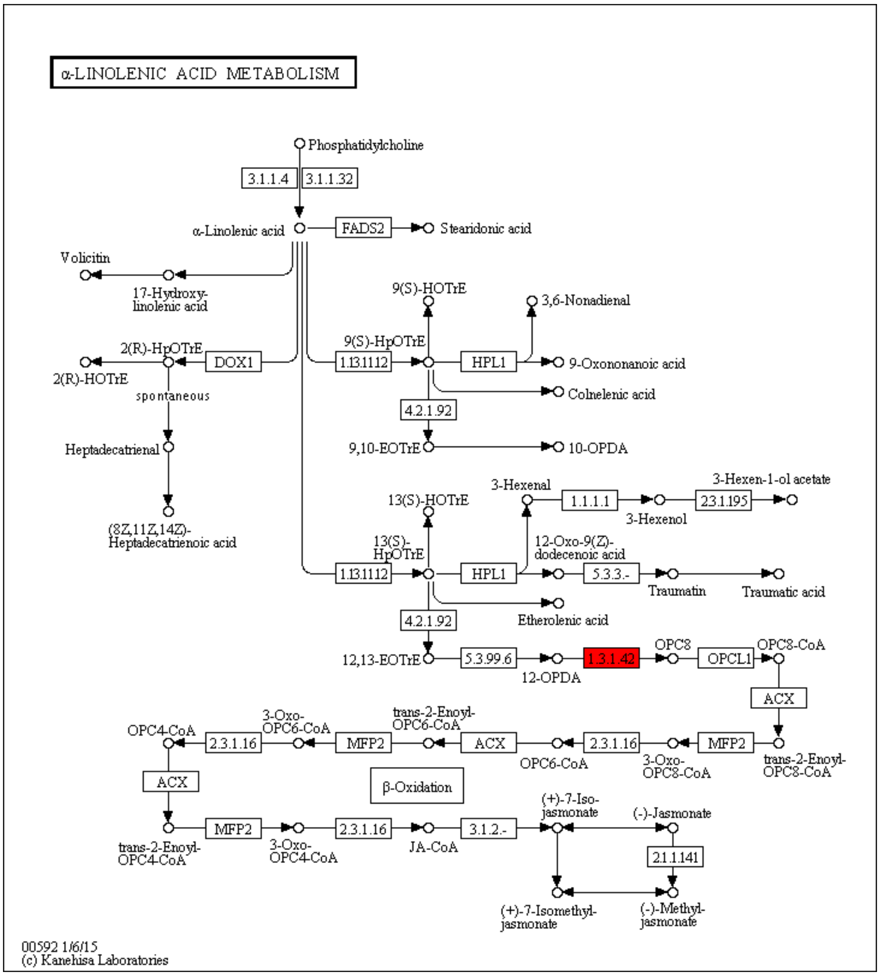

Figure S8 The expression of genes in the α-linolenic acid metabolism pathway (Ko00592) changed in response to DIECA application. The red color of 1.3.1.42 indicates that the expression of these genes was induced by DIECA application.

1.3.1.42 :TraesCS6B01G353200;TraesCS6D01G302300;TraesCS7A01G174700;TraesCS7B01G079500 (4.08850766740564;4.18241223233029;2.64911488226552;4.23336688954167)

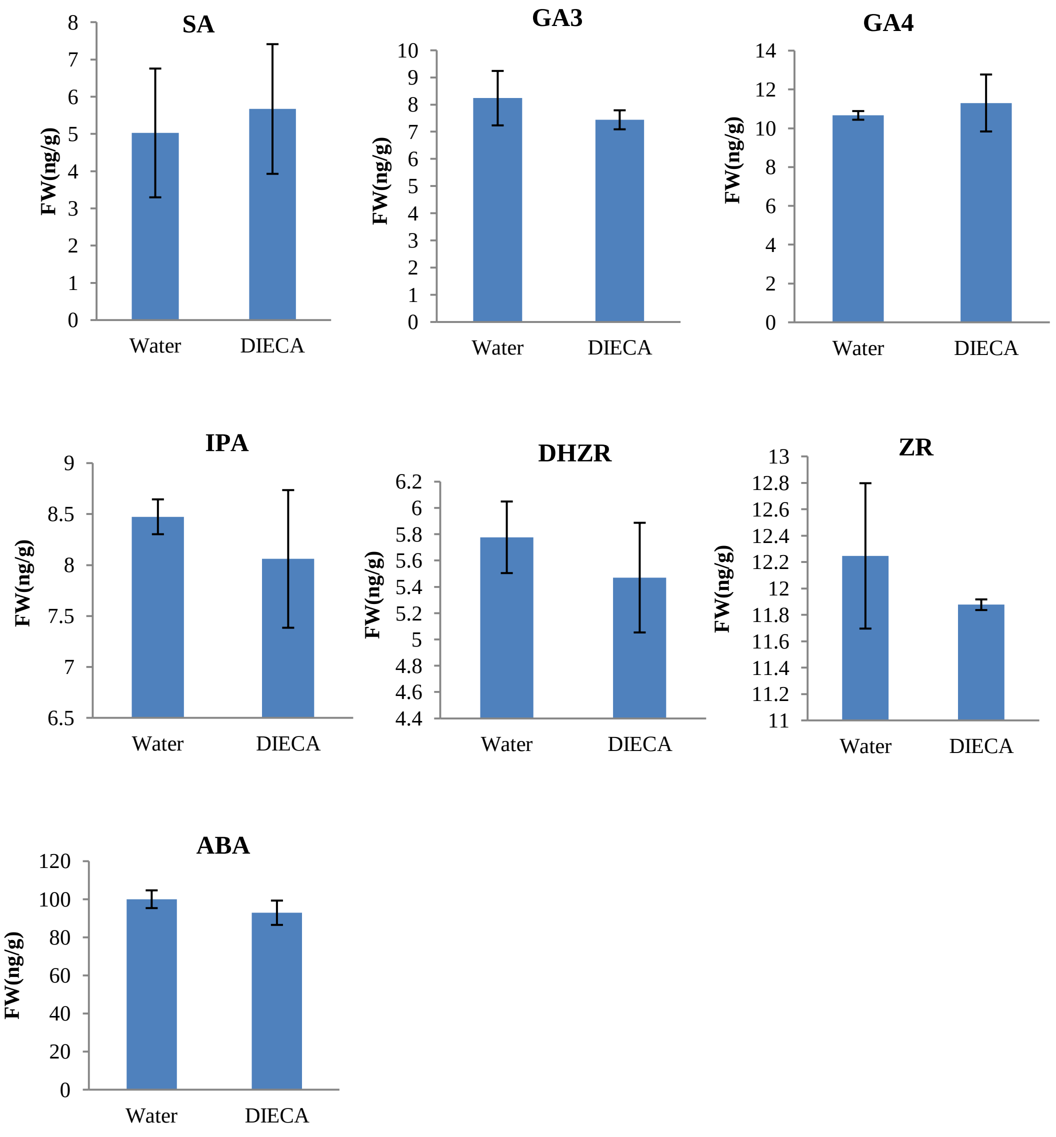

Figure S9 Content of SA, GA3, GA4, IPA, DHZR, ZR and ABA levels in water-treated and DIECA-treated Xuezao leaves. Each value is the mean ± SE of three independent biological repetitions. Asterisks indicate a significant difference from the water-treated mock control at $P \leq 0.05$ determined by Student’s t-test.

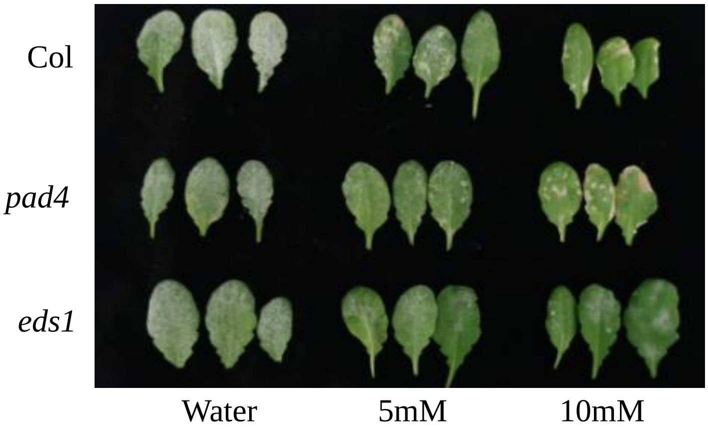

Figure S10 Morphology and resistance phenotypes of wild-type *Arabidopsis* Columbia (Col) and *pad4* and *eds1* mutant plants, pretreated with water, 5 mM DIECA, or 10 mM DIECA, at 10 dpi with UCSC1.

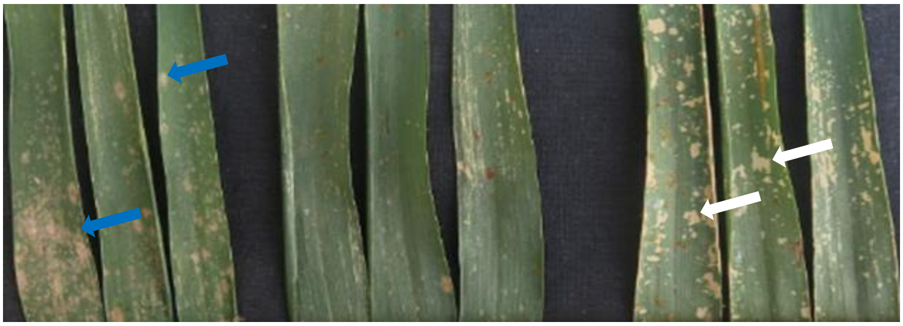

Water 5 mM DIECA 10 mM DIECA

Figure S11 Morphology and resistance phenotypes of Xuezao leaves with water, 5 mM and 10mM DIECA pretreatments in the artificial inoculation field in the 2017. DIECA treatments were taken 2 times (once every 5 days) prior to the outbreak period of powdery mildew disease. Images were taken at 5 days post second time of water and DIECA treatments. Powdery mildew disease signs are indicated by blue arrows. Necrotic spots are indicated by white arrows.
