# Supplementary material for: Exogenous sodium diethyldithiocarbamate, a Jasmonic acid biosynthesis inhibitor, induced resistance to powdery mildew in wheat
Source: Plant Direct. 2020 Apr 9;4(4):e00212. doi: 10.1002/pld3.212 (PMC7146025; doi:10.1002/pld3.212)
Supplement: Supplementary file 2 — Table S1 [file PLD3-4-e00212-s002.docx]

Table S1 The germination and penetration rates of powdery mildew in untreated, water-treated, and 10 mM DIECA-treated leaves at 24, 48, 96 and 120 hpi.

| Time (dpi) | Untreated | | Water-treated | | 10 mM DIECA-treated | |
| --- | --- | --- | --- | --- | --- | --- |
|  | Germination rate (%) | Penetration rate (%) | Germination rate (%) | Penetration rate (%) | Germination rate (%) | Penetration rate (%) |
| 24 h | 94.0±1.8 | 0 | 92.3.9±2.3 | 0 | 0** | 0 |
| 48 h | 99.0±1.2 | 64.9±4.5 | 99.0±1.0 | 71.3±5.1 | 6.7±1.8** | 0** |
| 96 h | 100 | 91.7±0.8 | 100 | 93.3±2.1 | 12.3±2.5** | 0** |
| 120 h | 100 | 100 | 100 | 100 | 29.9±1.9** | 5.6±0.4** |

Note: Germination rate (number of germinated spores relative to the total number of spores); Penetration rate (number of penetration spores relative to the total number of spores). Each value is the mean ± SE of three independent biological repetitions. Asterisks (**) indicate a significant difference from the untreated sample at P ≤ 0.01 determined by Student's t-test.
