# Supplementary material for: Exogenous sodium diethyldithiocarbamate, a Jasmonic acid biosynthesis inhibitor, induced resistance to powdery mildew in wheat
Source: Plant Direct. 2020 Apr 9;4(4):e00212. doi: 10.1002/pld3.212 (PMC7146025; doi:10.1002/pld3.212)
Supplement: Supplementary file 3 — Table S2 [file PLD3-4-e00212-s003.docx]

| **Samples** | **BMK-ID** | **Clean reads** | **Clean bases** | **GC Content** | **% ≥Q30** |
| --- | --- | --- | --- | --- | --- |
| DIECA-treated-1 | D01 | 30,122,509 | 8,998,902,274 | 56.79% | 90.50% |
| DIECA-treated-2 | D02 | 30,778,808 | 9,194,248,060 | 57.77% | 89.56% |
| DIECA-treated-3 | D03 | 28,557,220 | 8,535,083,130 | 57.09% | 89.47% |
| Water-treated-1 | H01 | 27,187,946 | 8,118,052,620 | 57.75% | 90.39% |
| Water-treated-2 | H02 | 25,749,518 | 7,688,191,412 | 57.96% | 90.29% |
| Water-treated-3 | H03 | 28,454,208 | 8,505,275,154 | 56.86% | 90.12% |

Table S2 Summary of the sequence data for the DIECA- and water-treated leaf samples.
