# Supplementary material for: Exogenous sodium diethyldithiocarbamate, a Jasmonic acid biosynthesis inhibitor, induced resistance to powdery mildew in wheat
Source: Plant Direct. 2020 Apr 9;4(4):e00212. doi: 10.1002/pld3.212 (PMC7146025; doi:10.1002/pld3.212)
Supplement: Supplementary file 4 — Table S3 [file PLD3-4-e00212-s004.docx]

| **BMK-ID** | **Total Reads** | **Mapped Reads** | **Unique Mapped Reads** | **Multiple Mapped Reads** | **Reads Mapped to '+'** | **Reads Mapped to '-'** |
| --- | --- | --- | --- | --- | --- | --- |
| D01 | 60,245,018 | 50,759,712 (84.26%) | 42,348,744 (70.29%) | 8,410,968 (13.96%) | 23,757,463 (39.43%) | 23,765,779 (39.45%) |
| D02 | 61,557,616 | 50,867,160 (82.63%) | 43,080,340 (69.98%) | 7,786,820 (12.65%) | 24,089,321 (39.13%) | 24,095,950 (39.14%) |
| D03 | 57,114,440 | 47,530,744 (83.22%) | 39,338,709 (68.88%) | 8,192,035 (14.34%) | 22,242,255 (38.94%) | 22,234,920 (38.93%) |
| H01 | 54,375,892 | 45,492,884 (83.66%) | 39,110,535 (71.93%) | 6,382,349 (11.74%) | 21,724,370 (39.95%) | 21,716,916 (39.94%) |
| H02 | 51,499,036 | 43,235,100 (83.95%) | 37,380,927 (72.59%) | 5,854,173 (11.37%) | 20,696,110 (40.19%) | 20,679,078 (40.15%) |
| H03 | 56,908,416 | 47,436,942 (83.36%) | 40,376,295 (70.95%) | 7,060,647 (12.41%) | 22,447,093 (39.44%) | 22,448,350 (39.45%) |

Table S3 The reads mapped to the wheat genome reference sequence.
