# Supplementary material for: Exogenous sodium diethyldithiocarbamate, a Jasmonic acid biosynthesis inhibitor, induced resistance to powdery mildew in wheat
Source: Plant Direct. 2020 Apr 9;4(4):e00212. doi: 10.1002/pld3.212 (PMC7146025; doi:10.1002/pld3.212)
Supplement: Supplementary file 5 — Table S4 [file PLD3-4-e00212-s005.docx]

| Annotation Database | Number of Annotated Genes | 300 bp≤length<1000 bp | length≥1000 bp |
| --- | --- | --- | --- |
| COG_Annotation | 34769 | 10109 | 24181 |
| GO_Annotation | 80428 | 29319 | 48088 |
| KEGG_Annotation | 33460 | 12384 | 19980 |
| KOG_Annotation | 52228 | 17236 | 33984 |
| Pfam_Annotation | 90176 | 32276 | 55356 |
| Swiss-Prot_Annotation | 73358 | 24795 | 46521 |
| eggNOG_Annotation | 109727 | 41676 | 63210 |
| NR_Annotation | 117985 | 46000 | 65753 |
| All_Annotated | 118189 | 46096 | 65819 |

Table S4 The number of annotated genes identified in searches of eight databases.
