# Supplementary material for: Exogenous sodium diethyldithiocarbamate, a Jasmonic acid biosynthesis inhibitor, induced resistance to powdery mildew in wheat
Source: Plant Direct. 2020 Apr 9;4(4):e00212. doi: 10.1002/pld3.212 (PMC7146025; doi:10.1002/pld3.212)
Supplement: Supplementary file 8 — Reviewers202028 [file PLD3-4-e00212-s008.pdf]

1. The time course of microscopic observations of fungal development is not consistent. In line 130 and in Table S1, the authors mentioned "at 24, 48, 96, and 120 h post-infection", while in line 365 and in the legend of Table S1, it showed "at 12, 24, 48, and 96 h post-powdery mildew infection". In addition, "hpi" appeared in the legend of Table S1, while "dpi" appeared inside the Table S1. Moreover, the supporting figure legends in the manuscript is different from which in the supporting supplemental files, such as Figure S1, please check carefully which one is the right one.

The time points are: 24, 48, 96 and 120 h. We have corrected them in line 127 and in Table S1.

As suggested we have carefully checked and corrected all the hpi and dpi in the MS.

We have modified legends of Figure S1, and checked all the legends in the supporting supplemental files.

We deleted the information of supporting figure legends in the MS, which should be inside of supporting supplemental files.

2. Grammar mistakes and unclear statements exist in several sentences and need to be corrected. For example, in line 32 "This treatment caused resistance to powdery mildew (Bgt) when applied prior to Bgt infection." It is not clear what "this treatment" refer to, and there is no subject after the "when"; in line 105-107 "We showed here that application of DIECA ...." The sentence is not clearly expressed and needs to be revised.

We have modified this sentence in line 26-28: “Our results showed that application of 10 mM DIECA 0-2 days before inoculation, effectively induced resistance to powdery mildew (*Bgt*) in wheat”.

We have changed this sentence to that in lines 100-102: Our results showed that application of the DIECA, the inhibitor of JA biosynthesis, could induce resistance to *Bgt* in wheat, while exogenous MeJA did not.

We have corrected these similar mistakes which could be found in MS by tracking marker.

3. There are still some mistakes regarding citations. In line 80-81, Xiang et al. 2011 should be cited once instead of twice. In line 103-104, "et al" should be italic. Authors should carefully check the citations.

We have corrected these mistakes and checked all the citations in MS.

4. In line 230, I think this sentence should be changed to "Changes were not also observed...". In line 278, "Further" should be lower case. ...

We have corrected according to reviewer’s suggestion.
